# Efficacy of rTMS Combined with Cognitive and Language Training in People Living with Alzheimer’s Disease: A Systematic Review

**DOI:** 10.3390/brainsci14090891

**Published:** 2024-08-31

**Authors:** Eleni-Nefeli Georgopoulou, Anastasia Nousia, Maria Martzoukou, Nefeli K. Dimitriou, Ioannis Liampas, Lambros Messinis, Grigorios Nasios

**Affiliations:** 1 Department of Speech and Language Therapy, School of Health Sciences, University of Ioannina, 45332 Ioannina, Greece; e.georgopoulou@uoi.gr (E.-N.G.); nefelikdimitriou@gmail.com (N.K.D.); 2 Department of Speech and Language Therapy, University of Peloponnese, 24100 Kalamata, Greece; sialogo@gmail.com; 3 Laboratory of Neuropsychology and Behavioral Neuroscience, School of Psychology, Aristotle University of Thessaloniki, 54124 Thessaloniki, Greece; mmartzo@lit.auth.gr (M.M.); lmessinis@psy.auth.gr (L.M.); 4 Department of Neurology, Faculty of Medicine, School of Health Sciences, University of Thessaly, 41500 Larissa, Greece; liampasioannes@gmail.com

**Keywords:** rTMS, cognitive and language training Alzheimer, Alzheimer, language rehabilitation, cognitive training and rTMS

## Abstract

Repetitive transcranial magnetic stimulation (rTMS) is a non-invasive brain stimulation method that has been suggested as a possible treatment method for cognitive impairment in patients with Alzheimer’s Disease (pwAD), similar to multidomain cognitive training (CT). The effectiveness, however, of combining these techniques for pwAD remains controversial due to the variability in rTMS parameters, differences in CT protocol designs—many of which neglect the language domain—and the inclusion of patients at various stages of Alzheimer’s Disease (AD) and with different types of dementia. The current review aims to evaluate the cognitive benefits of combining rTMS with CT, including language training, for individuals with mild to moderate AD. An extensive literature search was conducted in PubMed, Google Scholar, and the Cochrane Library with relevant terms, resulting in nine studies with a total of 290 participants [190 in the Active Group (AG) and 100 in the Control Group (CG)]. The comprehensive review of the articles revealed that the combined treatment improved global cognitive function, as well as neurocognitive, neuropsychiatric, and quality of life in the AG. Nevertheless, these results should be interpreted cautiously, given the relatively small number of existing studies on this specific combination.

## 1. Introduction

Alzheimer’s disease (AD) is a leading neurodegenerative condition. It is linked to the buildup of abnormal proteins in the brain, including beta-amyloid plaques and tau tangles, which result in the loss of brain cells. AD is a form of dementia leading to cognitive deficits, personality and behavioral changes, and motor impairments [1]. AD is associated with a gradual decline in memory, cognitive abilities, attention, and executive functions, as well as deterioration in thinking and behavioral skills [2,3]. It also affects language, reasoning, social conduct, verbal and auditory naming, and the capacity to perform basic tasks, all of which are significantly impaired by the underlying neuro-degenerative mechanisms [4]. Available medications, such as cholinesterase inhibitors (donepezil, rivastigmine, and galantamine) [5] and the glutamate receptor antagonist memantine [6], are used for symptomatic treatment. These treatments, however, have limited efficacy, do not prevent disease progression, and are not well-tolerated by all patients [7,8,9].

Therefore, it is critically important to expand the range of therapeutic options available for treating AD. One such option is cognitive training (CT) programs, which involve structured tasks/exercises presented either via computer or by paper and pencil. These programs range in difficulty level and target specific cognitive domains. They have shown promising results in AD, with positive effects on global and individual-domain cognitive outcomes [10]. Such interventions seem to be much more effective in a multidomain rather than a single-domain design base [11]. The results are still suboptimal, but they have a limited long-term beneficial impact. It is worth noting that CT programs in AD are often designed primarily to enhance memory, while language and other executive functions are often neglected, sometimes not trained at all, or not properly assessed [12]. Furthermore, despite numerous studies asserting the inclusion of language training in their CT programs, none of them offered detailed information regarding the specific language domains targeted or the content of the language tasks employed [10].

Among the non-pharmacological treatment modalities that have emerged in recent years is repetitive transcranial magnetic stimulation (rTMS). This treatment uses rhythmic electromagnetic induction (magnetic pulses) to non-invasively stimulate the brain, modulating cortical excitability and neural activity [13,14]. These pulses are generated by a coil placed on the scalp and can modulate neural activity in targeted brain regions. rTMS is considered a safe, low-cost, and effective treatment for patients with AD. Additionally, it is a promising alternative for the treatment of certain prevalent non-cognitive symptoms among patients with AD, including depression [15], as well as for the improvement of cognitive and motor functions in otherwise healthy older adults [16]. In addition, the combination of rTMS with neurophysiological techniques such as motor-evoked potential (MEP) allows for the reliable and convenient measurement of cortical plasticity [17]. Recent studies have reported that rTMS may specifically improve global cognitive function, language performance, and memory in patients with AD (pwAD), especially at the mild or early stage of the disease [18].

To date, there are four reviews and meta-analyses exploring the beneficial effects of rTMS on cognitive function in AD [19,20,21,22,23]. These articles, however, vary in terms of rTMS stimulation parameters (i.e., single site, multisite, etc.) and include both studies utilizing rTMS alone and studies combining rTMS with CT. They lack analytical information about the CT training programs (specific tasks, domains, etc.), and it appears that the CT programs used did not always include language in the training sessions. Furthermore, these reviews and analyses did not focus on specific clinical groups, as they analyzed studies examining patients with Mild Cognitive Impairment (MCI) as well as those with different stages of AD and other types of dementia.

Considering the well-established effects of rTMS and CT (with or without language included) on AD [24,25], we speculated that the combination of rTMS and CT but with language included might produce additional beneficial effects. Therefore, and due to the aforementioned literature gap, we conducted a systematic review carefully designed to summarize evidence from studies that implemented CT but specifically included language in the training, along with multisite rTMS for pwAD exclusively. Furthermore, analytical information regarding their CT programs is provided. In particular, the objectives of the present systematic review are as follows: the present systematic review aimed to (a) explore the effectiveness of combining high-frequency rTMS with CT, including language for patients with mild to moderate AD, and (b) determine which domains benefit most from the intervention.

## 2. Method

### 2.1. Search Strategy

A comprehensive literature search was performed using PubMed, Google Scholar, and the Cochrane Library. The following search terms were employed (entered as free text): (a) “rTMS cognitive and language training” and “Alzheimer”; (b) “Alzheimer” and “language rehabilitation” and “cognitive training” and “rTMS”. The final literature search was performed on 30th January 2024. The initial search yielded a total of 1538 studies published up to January 2024. Specifically, 546 studies were retrieved from PubMed, 848 studies from Google Scholar, and 144 studies from Cochrane.

### 2.2. Eligibility Criteria

**Figure 1:**
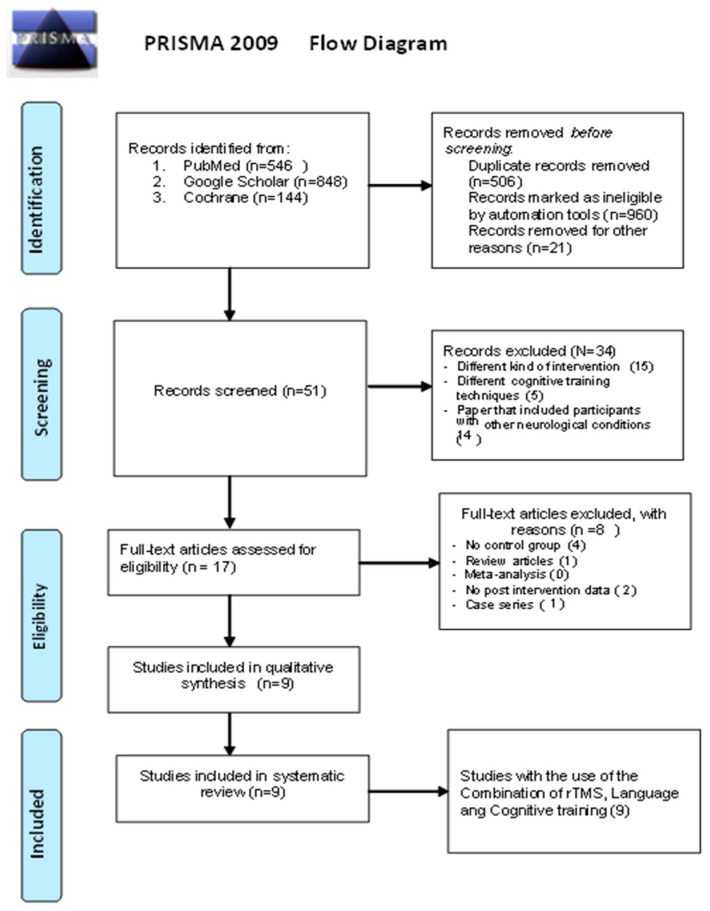
Caption [27,28,29,30,31,32,33,34,35].

The following eligibility criteria were considered: (1) use of rTMS combined with CT including language, (2) inclusion of participants with AD, (3) availability of pre- and post-intervention cognitive data, and (4) inclusion of at least 8 participants in the rTMS combined with CT (including language) arm. Studies were excluded if they (1) lacked pre- or post-intervention cognitive data, (2) involved different kinds of interventions (e.g., physical training, CT not including language, rTMS without CT including language), (3) included participants with other neurological conditions (e.g., stroke, Parkinson’s disease), or (4) were systematic review articles and meta-analyses. Conclusions were based on randomized controlled trials (RCTs). Further information regarding the procedure of the selection for eligible studies is presented in Figure 1 [26].

### 2.3. Data Extraction

The data listed below were exported from the retrieved studies: author, year of publication, number of participants, AD stage, targeted domains for intervention, study design, outcome measures, intervention duration and frequency, and outcomes. The PRISMA guidelines for reporting systematic reviews were followed (Appendix A) [26]. The flow diagram illustrates the progression of information through the different phases of the systematic review, mapping the number of records identified, included, and excluded, as well as the reasons for exclusions [36]. Two independent reviewers (A.N. and N.G.) conducted the search of the literature, performed the quality evaluation, and independently extracted the data. Any potential discrepancies were resolved by a third author (I.L.).

### 2.4. Risk of Bias Assessment Tool

**Figure 2:**
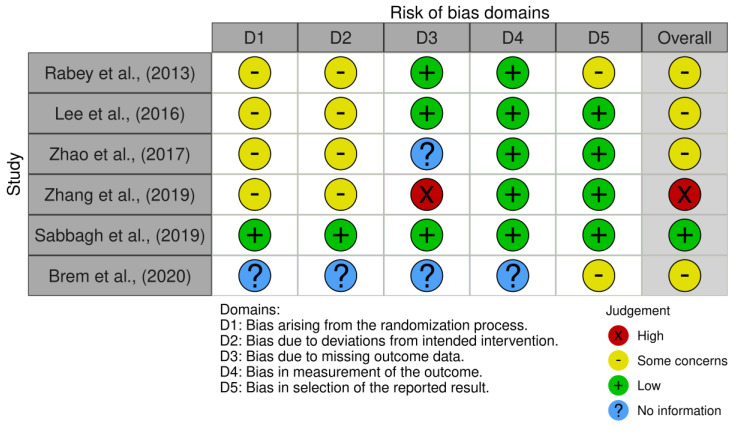
Caption [28,30,31,32,33,34].

Risk of bias was evaluated using the risk of bias (RoB) tool from Cochrane for systematic reviews of interventions (Figure 2). Five methodological domains were assessed: (1) randomization process (including sequence generation, allocation concealment, and baseline differences between groups), (2) deviations from intended interventions (such as blinding of participants and personnel, and appropriateness of analysis), (3) missing outcome data (data availability and reasons for missing data), (4) measurement of outcomes (methods of measurement for both groups), and (5) selective reporting (prespecified protocols and multiple analyses). Each domain was categorized as “low risk of bias”, “high risk of bias”, or “some concerns” based on the methodological features and reporting of the reviewed studies.

## 3. Results

### 3.1. Study Characteristics

Study characteristics are included in Table 1. Regarding the study settings, three studies were conducted in Israel [27,28,29], one study in both Israel and the USA [30], one study in Italy and the USA [31], one study in North Korea [32], two studies in China [33,34], and one study in France [35]. Two of them were pilot studies [27,33], and one was a clinical trial [29]. Three studies were randomized, double-blinded, and placebo-controlled [30,31,32,33]. One was randomized and double-blinded without placebo control [28]; one was randomized, single-blind, and placebo-controlled [31]; and, finally, one was a non-randomized open-label study [34].

Six of the studies [28,30,31,32,33,34] included both an active group (AG) and a sham group (SG), whereas all participants in the other three studies [27,29,35] received active stimulation. In total, 290 participants were included, with 190 in the AG and 100 in the SG. The treatment group received rTMS sessions combined with computer-based CT, including language in all studies except for one [34], which did not specify whether the training was computer-based or paper-pencil.

### 3.2. Participants’ Characteristics

Patients’ characteristics are presented in Table 2. In total, 290 patients were included. More specifically, the AG consisted of 190 individuals, 100 males and 90 females, while the SG included 100 participants, 52 males and 48 females [27,28,29,30,31,32,33,34,35]. The mean age of the participants ranged from 67.5 tο 76.9 years old; Rabey and Dobronevsky [29] did not provide information about the age of the participants. There were no significant differences in age and gender between the patient groups in the studies of Brem et al. [31], Lee et al. [32], Rabey et al. [28], Sabbagh et al. [30], Zhang et al. [33] and Zhao et al. [34], whereas Rabey and Dobronevsky [29] did not give specific information regarding the comparison in age and gender between groups. The participants in the other two studies [27,35] were not separated into groups.

In terms of disease severity, all nine studies included patients who were classified as having mild to moderate disease [27,28,29,30,31,32,33,34,35]. Five studies used the Diagnostic and Statistical Manual of Mental Disorders, 4th Edition (DSM-IV) criteria [27,28,30,32,34] to evaluate patients’ disease stage, and one study used the 5th edition’s criteria (DSM-5) combined with the National Institute on Aging and Alzheimer’s Association (NIA-AA) criteria [31].

One study fulfilled the National Institute of Neurological and Communicative Disorders and Stroke and the Alzheimer’s Disease and Related Disorders Association (NINCDS-ADRDA) criteria [33], and two studies provided no further information about the diagnostic process [29,35]. Moreover, five of the studies [27,28,29,32,34] supported the diagnosis with magnetic resonance imaging (MRI), and one study [32] used MRI to exclude cerebrovascular disease.

As a measure of baseline cognitive function, seven of the studies presented participants’ Mini Mental State Examination (MMSE) scores. Specifically, in five studies [27,28,30,31,32], the MMSE score ranged from 18 to 24; in one study [34], the score ranged from 18 to 26, and one study [29] did not provide the exact score. In addition, five studies used the Clinical Dementia Rate (CDR) scale; in two of these studies [27,28], the CDR score was 1, and in the other three studies [30,32,34], the CDR score was either 1 or 2. Moreover, Rabey and Dobronevsky [29] and Sabbagh et al. [30] used the Alzheimer Disease Assessment Scale—Cognitive (ADAS-Cog). Finally, Zhang et al. [33] conducted a baseline neuropsychological evaluation using CDR, MMSE, activities of daily living (ADL), and the Neuropsychiatric Inventory (NPI).

Three of the studies did not provide details about the concomitant pharmacological treatment administered to patients [27,29,35]. While Rabey et al. [28] noted that patients did not have serious metabolic or cardiac diseases, Zhao et al. [34], both with Lee et al. [32], reported no drug changes two months before and throughout the treatment duration, and Sabbagh et al. [30] and Zhang et al. [33] mentioned that if some patients were medicated for AD, they should have been taking the medication for at least three months at a stable dosage.

### 3.3. Intervention Characteristics

In all studies, participants underwent treatment programs with repetitive transcranial magnetic stimulation (rTMS) and CT, including language, which lasted for a period ranging between four weeks and three months, with session frequency varying from once per day to twice per week. Each session lasted from 30 to 60 min. The Neuro AD Neuronix protocol was applied in all studies except for two [33,34].

The majority of the studies stimulated six brain areas. Specifically, in most of the studies [27,28,29,30,31,32,35], the coil was placed on the Broca area (left frontal part of the temporal lobe), the Wernicke area (left posterior part of the temporal lobe), the right and left dorsolateral prefrontal cortex (R-dlPFC and L-dlPFC), and the right and left parietal somatosensory association cortex (R-pSAC + L-pSAC). In one study [34], the coil was placed on the parietal P3/P4 and the posterior temporal T5/T6 areas, and in another study [33], it was placed on the DLPFC and the lateral temporal lobe (LTL). Tasks were designed to train syntax, grammar, lexical meaning, categorization, action naming, object naming, spatial memory, and spatial attention. All tasks were associated with the stimulation of the corresponding brain areas. Analytical information is shown in Table 3.

### 3.4. Cognitive Including Language Outcomes

The targeted domains were language functions, judgment, executive function, long-term memory, spatial and topographical orientation, and praxis in almost all studies [27,28,29,30,31,32,35], except for the studies of Zhao et al. [34], who targeted memory, language and executive function, and Zhang et al. [33], who aimed at the domains of memory, mathematical calculations, language, and logical thinking.

The comprehensive review of the articles revealed the benefits of rTMS and CT, including language treatment, which are summarized in Table 4. Two studies [29,30] used only the Alzheimer Disease Assessment Scale—Cognitive (ADAS-cog) to evaluate the outcomes of the therapy sessions; both showed a statistically significant benefit favoring the active group over the sham group. Four studies [27,28,31,32] evaluated the language and cognitive profile before and after treatment with the ADAS-cog and the Mini Mental State Examination (MMSE). Specifically, Rabey and Dobronevsky [29] found statistically significant improvement in both tests after the combination of rTMS CT, including language treatment, whereas patients in the studies of Bentwich et al. [27] and Lee et al. [32] showed significant improvement only in ADAS-cog. In the latter research, however, the MMSE score improved significantly between baseline and six weeks after treatment in the mild AD group. Brem et al. [31] noticed that the group with active stimulation and real CT, including language treatment, showed significant improvement compared to the sham stimulation and sham CT/language group but not compared to the real CT/language and sham stimulation group.

The other three studies [33,34,35] assessed participants’ outcomes with additional tests. In particular, Zhao et al. [34] found significantly improved performance in the ADAS-cog; the MMSE; and the World Health Organization University of California—Los Angeles, Auditory Verbal Learning Test (WHO-UCLA AVLT) score for the AG, with notably improved Montreal Cognitive Assessment (MoCA) scores in mild AD patients of the AG. Nguyen et al. [35] used the ADAS-cog, the MMSE, the Stroop color test, and Dubois’ five-word testing (5WT), of which only the ADAS-cog indicated a significantly improved score. Finally, Zhang et al. [33] reported improved scores in the ADAS-cog (specifically in the word recall memory subscale), the MMSE, and Addenbrooke’s Cognitive Examination III (ACE-III, in the domains of attention, memory, visual–spatial function), with these effects lasting for at least four weeks.

### 3.5. Neurocognitive, Neuropsychiatric, and Quality of Life Outcomes

Apart from language and cognitive domains, seven studies [27,28,30,31,32,33,35] also evaluated non-cognitive factors. Three of these studies [27,31,33] assessed the impact of the treatment on patients’ quality of life. For this purpose, Bentwich et al. [27] and Brem et al. [31] used the Alzheimer Disease Assessment Scale—Activities of Daily Living (ADAS-ADL) with positive outcomes, and Zhang et al. [33] demonstrated a tendency toward improvement in the Activities of Daily Living (ADL). Depression was measured either with the Hamilton Rating Scale for Depression [27] or with the Geriatric Depression Scale (GDS) [31,32]. The study by Bentwich et al. [27] showed an improved score but without statistical significance, whereas the study by Lee et al. [32] found that the GDS score did not improve significantly in the treatment group. Brem et al. [31] evaluated depression only at baseline.

Five of the studies [27,28,30,31,32] used the Clinical Global Impression of Change scale (CGIC), all of which indicated better results. In addition, three studies assessed the presence and severity of neuropsychiatric symptoms using the Neuropsychiatric Inventory test (NPI); two of them [28,33] found an improvement after treatment, while the other one [27] noted that the NPI scores did not change after treatment. Moreover, two studies [31,33] assessed patients using the Clinical Dementia Rating (CDR) at the baseline phase. Lastly, Nguyen et al. [35] utilized the Frontal Assessment Battery (FAB), locomotor, apathy score, Zarit score, and dependence score, showing an improvement in apathy and dependence scores. His section may be divided by subheadings. It should provide a concise and precise description of the experimental results, their interpretation, and the experimental conclusions that can be drawn.

## 4. Discussion

To the best of our knowledge, the present review is the first to focus on the impact of combining rTMS with CT, including language, for pwAD, on their overall language and cognitive profile. The critical examination of the retrieved studies revealed that this combinatory treatment led to language and cognitive improvement as assessed with the ADAS-Cog, WHO-UCLAAVLT, MMSE, MoCA, and ACE-III. In contrast, using rTMS or CT, including language alone, did not result in notable improvements.

Our findings are consistent with those of Cheng et al. [37], who concluded in their meta-analysis that combining rTMS with CT enhances cognitive abilities in Alzheimer’s disease more effectively than rTMS alone. It should be noted, however, that their analysis included studies where CT did not necessarily train language along with other cognitive domains. Furthermore, Lin et al. [19], in their review, did not reveal significantly improved results when combining rTMS with CT but observed that the complementary use of CT yielded greater overall enhancement than either treatment alone. Similar findings were observed in the reviews and meta-analyses by Xie et al. [20], Wei et al. [21], and Liu [23]. In contrast to our findings, Yan et al. [38] found in their systematic review and meta-analysis that the additional application of CT did not show significant improvement in the cognitive status of the participants. Their sample, however, included both Mild Cognitive Impairment (MCI) participants and individuals with different stages of pwAD.

Specifically, beneficial outcomes were observed for language and overall cognitive profile [28,31,32,33,34,35], as well as for memory and overall cognitive profile [27,32,33,34], and for quality of life [27,31,33]. Furthermore, outcomes regarding neuropsychiatric symptoms and quality of life were conflicting and lacked statistical significance. Specifically, while quality of life outcomes showed a tendency towards improvement [27,31,34], improvements in neuropsychiatric symptoms did not reach significance [27,28,35]. It is worth noting that all studies reported beneficial effects for their active groups on overall cognitive profile [27,28,29,30,31,32,33,34,35].

The studies that provided stronger evidence were primarily those offering outcome data for both language and executive functions [28,31,32,33,34,35]. These results, however, should be interpreted cautiously due to several factors. Firstly, participant nationality varied across the studies, leading to potential differences in language tasks. Additionally, sample sizes varied, further influencing the robustness of the findings. Moreover, there remains an insufficient number of studies focusing on pwAD and the impact of combining rTMS with CT, including language intervention, to draw definite conclusions. Another significant limitation is the lack of comprehensive analytical evaluation beyond the language sections of assessment tools such as ADAS-Cog and ACE-III, both before and after interventions, which hampers the ability to assess the specific impact on the language domain distinct from other cognitive and executive functions.

It has been suggested that combining rTMS with CT merges “exogenous” and “endogenous” stimulation, thus enhancing neuroplasticity. rTMS may pre-activate the neural system, while subsequent CT interacts with the ongoing brain activation to amplify or extend the neural effects [39,40,41]. This synergistic interaction may allow CT to modulate the impact of rTMS, potentially explaining why studies involving both rTMS and CT predominantly yield more consistent results than those without cognitive training. Multidomain or holistic CT, including language, would be the optimal approach. This review and the data derived from the aggregation of the results of the included studies demonstrate originality, as it is the first time the effectiveness of rTMS combined with CT and language training has been investigated. Additionally, it contributes to the current literature and holds significant clinical relevance, as the role of language (and its training) in AD seems to be critically important and should always be taken into consideration.

Furthermore, our review fully supports the relevance of non-pharmacological multidomain interventions for pwAD and provides the groundwork for future studies to build upon. When it comes to the effectiveness of these interventions, there is growing evidence (supported even by the present review) that a multidomain design, which targets a variety of domains and brain regions, seems to be more efficient and effective [11,42,43,44,45]. Many questions, however, remain to be answered, including how language and other cognitive improvements would translate into improved patients’ quality of life and whether neurophysiological measures could act as potential biomarkers for designing interventions in AD. Future research should assess in greater detail the potential that language rehabilitation might play a more important role in the improvement of the overall everyday living profile of patients and their caregivers/families.

## Figures and Tables

**Table 1:** General information of the retrieved studies.

Study	Country	NIBS	Study Design	Parallel Arm	Groups	Participants per Group
Bentwich et al. (2011) [27]	Israel	rTMS/Computer CT, including language	Pilot	-	AC	8
Rabey et al. (2013) [28]	Israel	rTMS/Computer CT, including language	Randomized, double-blind	YES	AG	7
					SG	8
Lee et al. (2016) [32]	North Korea	rTMS/Computer CT, including language	Randomized, double-blind, placebo-controlled	YES	AG	18
					SG	8
Rabey and Dobronevsky (2016) [29]	Israel	rTMS/ Computer CT, including language	Clinical trial	YES	AG	25
					AG2	5
Zhao et al. (2017) [34]	China	rTMS/ CT, including language	Prospective randomized, double-blind, placebo-controlled trial	YES	AG	17
					SG	13
Nguyen et al. (2017) [35]	France	rTMS/Computer CT, including language	Prospective open-label	-	AG	10
Sabbagh et al. (2019) [30]	USA + Israel	rTMS/Computer CT, including language	Prospective randomized, double-blind, sham-controlled	YES	AG	59
					SG	50
Zhang et al. (2019) [33]	China	rTms/ Computer CT, including language	Pilot	YES	AG	15
					SG	13
Brem et al. (2020) [31]	USA + Italy	rTMS/Computer CT, including language	Randomized, sham controlled	YES	AG1	16
					AG2	10
					SG (Sc + ST)	8

CT: Cognitive training; rTMS: repetitive transcranial magnetic stimulation; AG: active group; SG: sham group.

**Table 2:** Demographics of study participants of the retrieved studies.

Study	N	AD Stage	AG1(r/r)		AG2(r/s)		SG(s/s)	
			Gender (Males/Total)	Mean Age in Years (Sd)/Range in y	Gender (Males/Total)	Mean Age in y (Sd)/Range in Years	Gender (Males/Total)	Mean Age in y (Sd)/Range in Years
Bentwich et al. (2011) [27]	8	Mild to moderate	7/8	75.4 (4.4)	--	--	--	--
Rabey et al. (2013) [28]	15	Mild to moderate	5/7	72.6 (8.9)	--	--	5/8	75.4 (9.07)
Lee et al. (2016) [32]	26	Mild	8 /18	72.1 (7.6)	--	--	3/ 8	70.3 (4.8)
Rabey and Dobronevsky (2016) [29]	30	Mild to moderate	17/30	N/A	N/A/5	N/A	--	--
Zhao et al. (2017) [34]	30	Mild to moderate	7/17	69.3 (5.8)	--	--	7/13	71.4 (5.2)
Nguyen et al. (2017) [35]	10	Mild	5/10	73 (7.2)	--	--	--	--
Sabbagh et al. (2019) [30]	109	Mild to moderate	41/59	76.9 (NA)	--	--	29/50	76.7 (NA)
Zhang et al. (2019) [33]	28	Mild	3/15	69 (8.19)	--	--	3/13	68.54 (7.93)
Brem et al. (2020) [31]	34	Mild to moderate	4/16	69.25 (6.80)	5/10	69.10 (5.24)	5/ 8	67.50 (10.27)

N: number of participants; AD: Alzheimer’s Disease; AG: active group; SG: sham group; AG2: 2nd active group; Y: years; Sd: standard deviation; N/A: not available.

**Table 3:** Summary of the procedural characteristics and main findings of the retrieved studies.

Study	N	Coil Placement	Diagnostic Criteria/Inclusion Criteria	Cognitive Domains Targeted	Stimulation Parameters	Number of Sessions	Outcome Measures	Results
Bentwich et al. (2011) [27]	8	Neuro AD Neuronix Broca (left frontal part of the temporal lobe) Wernicke (left posterior part of the temporal lobe) R-dlPFC + L-dlPFC (right and left prefrontal cortex) R-pSAC + L-pSAC (right and left parietal somatosensory association cortex)	DSM-IV criteria MMSE score18–24 CDR (1) Hebrew language MRI supporting probable AD Having a caregiver	Language functions Judgment Executive function Long-term memory Spatial and topographical orientation and praxis	45 min/session 3 brain areas stimulated/session 20 trains of rTMS (2 s of 10 Hz/train, 20 pulses/train.) Followed by 1–4 cog. tasks during a period of 20–40 s 20 repetitions = Each area stimulated with 400 pulses during a 7–15 min.	Int-rTMS-COG 1/day for 6 weeks = 30 sessions and maint-rTMS-COG 2/week for 3 months = 24 sessions 54 total	ADAS-cog CGIC MMSE ADAS-ADL HAMILTON NPI	ADAS-Cog (average) improved by approximately 4 points after both 6 weeks and 4.5 months of treatment and CGIC by 1.0 and 1.6 points, respectively. MMSE, ADAS-ADL, and HAMILTON improved, but without statistical significance. NPI did not change.
Rabey et al. (2013) [28]	15	Neuro AD Neuronix Broca (left frontal part of the temporal lobe) Wernicke (left posterior part of the temporal lobe) R-dlPFC + L-dlPFC (right and left prefrontal cortex) R-pSAC + L-pSAC	DSM-IV criteria MMSE score18–24 CDR (1) MRI indicating probable AD No serious metabolic or cardiac diseases Having a caregiver at least for 10 h/week Hebrew or Russian language	Language functions Judgment Executive function Long-term memory Spatial and topographical orientation and praxis	45–60 min/session 3 brain areas stimulated/session 20–25 trains of rTMS (2 s of 10 Hz/region, 20 pulses/train.) Followed by 1–4 cog. tasks during a period of 20–40 s 20 repetitions = Each area stimulated with 400–500 pulses during a 7–15 min.	Int-rTMS-COG 1/day for 6 weeks = 30 sessions and maint-rTMS-COG 2/week for 3 months = 24 sessions 54 total	ADAS-cog CGIC NPI	There was an improvement in the average ADAS-cog score of 3.76 points after 6 weeks in the treatment group compared to 0.47 in the placebo group and 3.52 points after 4.5 months of treatment, compared to a worsening of 0.38 in the placebo. There was also an improvement in the average CGIC score of 3.57 (after 6 weeks) and 3.67points (after 4.5 months), compared to 4.25 and 4.29 in the placebo group (mild worsening). NPI improved non-significantly.
Lee et al. (2016) [32]	26	Neuro AD protocol Neuronix Broca (left frontal part of the temporal lobe) Wernicke (left posterior part of the temporal lobe) R-dlPFC + L-dlPFC (right and left prefrontal cortex) R-pSAC + L-pSAC (right and left parietal somatosensory association cortex	DSM-IV criteria MMSE score18–24 CDR(1 or 2) Having a caregiver Read and write Korean MRI	Language functions Judgment Executive function Long-term memory Spatial and topographical orientation and praxis	60 min/session 3 brain areas stimulated/session 20 trains of rTMS (2 s of 10 Hz/train, 20 pulses/train.) Followed by 1–4 cog. tasks during a period of 20–40 s 20 repetitions = Each area stimulated with 400 pulses during a 7–15 min.	1/day for 6 weeks = 30 sessions	ADAS-cog CGIC MMSE GDS	The ADAS-cog score in the treatment group was significantly improved compared to the sham group (4.28 and 5.39 in the treatment group vs. 1.75 and 2.88 in the sham group immediately and 6 weeks after treatment, respectively). The MMSE and CGIC scores were also improved in the treatment group. Based on subgroup analysis, the effect of rTMS-COG was superior for the mild group compared to the total patients, especially in the domains of memory and language.
Rabey and Dobronevsky, (2016) [29]	30	Neuro AD Neuronix Broca (left frontal part of the temporal lobe) Wernicke (left posterior part of the temporal lobe) R-dlPFC + L-dlPFC (right and left prefrontal cortex) R-pSAC + L-pSAC (right and left parietal somatosensory association cortex	Mild to moderate AD diagnosis ADAS-Cog MMSE MRI	Language functions Judgment Executive function Long-term memory Spatial and topographical orientation and praxis	60 min/session 3 brain areas stimulated/session 20 trains of rTMS (2 s of 10 Hz/train, 400 pulses/train) for 3/4 paradigms and 5 trains (2 s of 10 Hz/train, 100 pulses/train) for the 4th paradigm	1/day for 6 weeks = 30 sessions Second treatment course for 5 participants after 10.2 months	ADAS-Cog MMSE	The effect of rTMS-COG treatment was statistically significant regarding both ADAS-Cog (−2.4 point improvement) and MMSE (1.7 points improvement) scores.
Zhao et al. (2017) [34]	30	Parietal P3/P4 Posterior temporal T5/T6	DSM-IV criteria MMSE 18–26 CDR 1 or 2 Caregiver for >72 h/week Capable of Reading and writing Chinese language MRI No drug change 2 months before and throughout the duration	Memory Language Executive function	1 h/session 3 brain areas each session 10 min of rTMS (10 s of 20 Hz/train, 20 s intermediate/train) followed by 2–4 cognitive tasks over the course of 20–40 s/brain area	1/day, 5days/week for 6 weeks = 30 sessions No maintenance intervention	ADAS-Cog MMSE MoCA WHO-UCLAAVLT	The ADAS-cog, MMSE, and WHO-UCLA AVLT score in the rTMS group was significantly improved compared with baselines at 6 weeks after treatment (all *p* < 0.05). Meanwhile, MoCA scores were also obviously ameliorated in the mild AD patients with rTMS. Additionally, subgroup analysis showed that the effect of rTMS on the memory and language of mild AD patients were superior to those of moderate AD patients.
Nguyen et al. (2017) [35]	10	Neuro AD Neuronix Broca (left frontal part of the temporal lobe) Wernicke (left posterior part of the temporal lobe) R-dlPFC + L-dlPFC (right and left prefrontal cortex) R-pSAC + L-pSAC (right and left parietal somatosensory association cortex	Probable AD diagnosis	Language functions Judgment Executive function Long-term memory Spatial and topographical orientation and praxis	30–45 min/session 3 brain areas stimulated/session 20 trains of rTMS (2 s of 10 Hz/ train, 20 pulses/train.) 20 repetitions = Each area stimulated with 400 pulses	1/day for 5 weeks= 25 sessions	ADAS-Cog MMSE Dubois FAB Stroop Locomotor Apathy score Zarit score Dependence score	The primary endpoint was the improvement of the ADAS-Cog score. Six months after the end of the treatment, the ADAS-Cog score returned to baseline value, except for the best responders, who remained significantly improved. The other main result was the improvement of apathy and dependence scores for the entire series of patients.
Sabbagh et al. (2019) [30]	109	Neuro AD Neuronix Broca (left frontal part of the temporal lobe) Wernicke (left posterior part of the temporal lobe) R-dlPFC + L-dlPFC (right and left prefrontal cortex) R-pSAC + L-pSAC (right and left parietal somatosensory association cortex	DSM-IV criteria MMSE score18–24 CDR(1 or 2) Age 60–90 ADAS-Cog >17 Reliable caregiver No deficits in hearing or vision Good English or Hebrew >8 th grade education Stable dose >90 days (if medicated for AD)	Language functions Judgment Executive function Long-term memory Spatial and topographical orientation and praxis	60 min/session 3 brain areas stimulated/session trains of rTMS (2 s of 10 Hz/train, 20 pulses/train.) 20 repetitions = Each area stimulated with 400 pulses	1/day, 5days/week for 6 weeks = 30 sessions	ADAS-Cog CGIC MRI	Subjects with baseline ADAS-Cog showed a statistically significant benefit favoring active over sham. Responder analysis showed 31.7% participants in the active group with ≤−4 point improvement on ADAS-Cog versus 15.4% in the sham group.
Zhang et al. (2019) [33]	28	DLPFC LTL	NINCDS-ADRDA Tests for: vitamin B12, folate, and thyroid hormone levels; quantitative hepatitis C antibody, quantitative human immunodeficiency virus antibody, and syphilis serology E epsilon 4 (APOEε4) genotype MRI to exclude cerebrovascular disease Before therapy, patients had taken the medicine for at least 3 months at a stable dosage. neuropsychological evaluation.	Memory Mathematical calculations Language Logic thinking	60 min/session 2 brain areas Repetition of 10 Hz for 5 s and intermittent for 25 s 20 trains = 1000 pulses Approximately 10 min/encephalic region	1/day, 5 days/week for 4 weeks 20 total No maintenance sessions	ADAS-Cog CDR MMSE ADL NPI ACE-III	After 4 weeks of rTMS-CT treatment, the patients’ scores improved in the ADAS-cog, MMSE, ACE-III, in the subscales of word recall memory in the ADAS-cog, and in the domains of attention, memory, visual–spatial function in the ACE-III. The effects lasted for at least 4 weeks
Brem et al. (2020) [31]	34	Neuro AD Neuronix Broca (left frontal part of the temporal lobe) Wernicke (left posterior part of the temporal lobe) R-dlPFC + L-dlPFC (right and left prefrontal cortex) R-pSAC + L-pSAC (right and left parietal somatosensory association cortex	DSM-V criteria NIA-AA Criteria MMSE score 18–24 Age 55–90 Normal/corrected ability to see and hear English or Italian as primary language	Language functions Judgment Executive function Long-term memory Spatial and topographical orientation and praxis	60 min/session 3 brain areas stimulated/session 20 trains of rTMS (2 s of 10 Hz/ region, 20 pulses/train.) Followed by 1–4 cog. tasks during a period of 20–40 s 15 repetitions/region	1/day for 6 weeks = 30 sessions	ADAS-cog GDS CDR ADAS-ADL MMSE CGIC	The real/real group showed significant cognitive improvement compared to the sham/sham, but not the real/sham group.

N: number of participants; MMSE: Mini Mental Stage Test; DSM-IV: Diagnostic and Statistical Manual of Mental Disorders, 4th Edition; DSM-V: Diagnostic and Statistical Manual of Mental Disorders, 5th Edition; rTMS: repetitive transcranial magnetic stimulation; CDR: Clinical Dementia Rating; ADAS-cog: Alzheimer Disease Assessment Scale—Cognitive; CGIC: Clinical Global Impression of Change scale; ADAS-ADL: Alzheimer Disease Assessment Scale—Activities of Daily Living; WHO-UCLAAVLT: World Health Organization university of California—Los Angeles, Auditory Verbal Learning Test; NIA- AA:National Institute on Aging—Alzheimer’s Association criteria; COG: Cognitive Training; Dubois: the 5 word test; Hamilton Rating Scale for Depression; MoCA: Montreal Cognitive Assessment NPI: Neuropsychiatric Inventory test; ICF, intracortical facilitation; LICI, long-interval intracortical inhibition; rMT/aMT, resting/active motor threshold; SICI, short-interval intracortical inhibition; GDS: Geriatric Depression Scale; FAB: Frontal Assessment Battery; Dubois score; Stroop color test; R-pSAC: parietal somatosensory association cortex; L-pSAC: left parietal somatosensory association cortex; R-dlPFC: right prefrontal cortex; L-dlPFC: left prefrontal cortex); ACE-III: Addenbrooke’s Cognitive Examination III; NINCDS-ADRDA: National Institute of Neurological and Communicative Disorders and Stroke—the Alzheimer’s Disease and Related Disorders Association criteria.

**Table 4:** Detailed results (neuropsychological measures, global cognitive function, and mood) from the studies.

Study	Outcome Measures	AG-PRE			AG-POST 6 Weeks					AG-POST 6 Weeks						
**Bentwich et al. (2011)** [27]		**Mean (Sd)**			**Mean (Sd)**				***p*-value**	**Mean (Sd)**			***p*-value**			
	**ADAS-Cog**	22.5 (5.6)			18.3 (6.8)				--	18.5 (3.8)			--			
	**CGIC**	3.0 (1.0)			2.4 (0.7)				--	N/A			--			
	**MMSE**	22.9 (1.7)			24.1 (2.2)				*p* = 0.049	22.1 (2.1)			--			
	**ADAS-ADL**	61.4 (14.6)			66.3 (15.1)				--	63.0 (17.8)			--			
	**HAMILTON**	9.7 (4.2)			5.6 (1.8)				*p* = 0.053	8.3 (2.7)			--			
	**NPI**	5.2 (7.8)			3.8 (8.9)				--	7.1 (10.0)			--			
**Rabey et al. (2013)** [28]		**AG-PRE**			**AG-POST 6 weeks**		**AG-POST 4.5 months**			**SG-PRE**		**SG-POST** **6 weeks**	**SG-POST** **4.5 months**			
		**Mean (Sd)**			**Mean (Sd)**		**Mean (Sd)**		***p*-value**	**Mean (Sd)**		**Mean (Sd)**	**Mean (Sd)**			***p*-value**
	**MMSE**	22(8.9)			--		--			22 (1.41)						
	**ADAS-Cog**	24.09			20.33		20.57		*p* = 0.04	24.09		23.19	24.47			*p* = 0.05
	**GCIC**				3.57		3.67		*p* = 0.05			4.25	4.29			*p* = 0.05
	**NPI**				+3.43							−1.38				
**Lee et al. (2016)** [32]		**Group (n)**			**Pre** **(Mean, Sd)**				**Post** **(Mean, Sd)**	**6weeks after** **(Mean, Sd)**			***p*-value**			
													**(Pre) vs. post**		**(Pre) vs. 6 week after**	**Time group**
	**ADAS-Cog**	**Treatment**			23.61 (6.40)				19.33 (8.30)	18.22 (8.85)			0.0018		0.002	0.208
		**Sham**			22.88 (6.20)				21.12 (7.66)	20.00 (9.49)			0.238		0.46	
		**Mild treatment**			21.77 (5.09)				16.31 (6.40)	14.92 (7.43)			0.035		0.005	0.111
		**Mild sham**			20.83 (3.76)				18.17 (4.54)	17.33 (4.93)			0.396		0.056	
		**Moderate treatment**			28.4 (7.54)				27.2 (7.95)	26.8 (6.30)			1		0.264	0.966
		**Moderate sham**			29.0 (9.90)				30.0 (9.90)	28.0 (18.39)			NA		NA	
	**MMSE**	**Treatment**			22.39 (2.87)				23.89 (4.44)	24.39 (4.57)			0.139		0.058	0.729
		**Sham**			22.75 (2.49)				24.50 (4.90)	25.75 (4.56)			0.769		0.213	
		**Mild treatment**			23.77 (2.01)				25.62 (3.33)	26.46 (2.93)			0.058		0.015	0.785
		**Mild sham**			23.83 (1.71)				26.67 (2.16)	27.5 (2.51)			0.461		0.204	
		**Moderate treatment**			8.8 (0.84)				19.4 (3.98)	19.0 (3.54)			1		1	0.784
		**Moderate sham**			19.5 (0.71)				18.0 (5.66)	20.5 (6.36)			NA		NA	
		**Treatment**			10.50 (6.41)				7.89 (5.71)	7.50 (6.44)			0.635		0.452	0.77
	**GDS**	**Sham**			11.63 (8.93)				9.38 (6.99)	8.00 (6.97)			0.656		0.020	
		**Mild treatment**			11.62 (4.82)				9.92 (5.25)	10.00 (5.85)			1		1	0.754
		**Mild sham**			11.83 (8.45)				9.17 (6.97)	7.50 (6.16)			1		0.148	
		**Moderate treatment**			7.60 (8.71)				2.60 (2.70)	1.00 (0.71)			1		1	0.484
		**Moderate sham**			11.00 (14.14)				10.00 (9.90)	9.50 (12.02)			NA		NA	
					**Changes in scores mean (SD)**					***p*-value**						
	**Group**	**Cognitive** **domains**			**Δ immediate after treatment**				**Δ 6 weeks after**	**Baseline vs. immediate after**			**Baseline vs. 6 weeks after**		**time**	
	**Treatment**	**Memory**			2.00 (2.81)				2.56 (3.05)	0.040			0.006		0.054	
		**Language**			1.28 (1.53)				1.44 (1.92)	0.004			0.003		0.009	
		**Executive function**			0.67 (1.24)				0.56 (1.50)	0.117			0.413		0.995	
	**Mild treatment**	**Memory**			1.77 (3.09)				2.69 (3.71)	0.275			0.031		0.178	
		**Language**			1.23 (1.79)				1.23 (1.96)	0.04			0.019		0.002	
		**Executive function**			0.92 (1.12)				0.92 (1.50)	0.031			0.093		0.2	
	**Moderate Treatment**	**Memory**			2.60 (2.07)				2.20 (3.03)	0.627			0.688		0.397	
		**Language**			1.40 (0.55)				2.00 (1.87)	0.243			1.000		0.947	
		**Executive function**			0.00 (1.41)				0.40 (1.14)	1.000			0.969		0.084	
**Rabey and Dobronevsky, (2016)** [29]	**Parameter/measurement**			**Mean**	**SE**		**Min**		**Median**	**Max**	**N**	***p*** **-value**				
	**ADAS-Cog**	**Pre-treatment**		**20.5**	1.3		10.6		18.8	37.3	30					
		**Post-treatment**		**18.1**	1.4		8.3		16.2	33.6	30					
		**Difference**		**-2.4**	0.6		--		--	--	30	<0.001				
	**MMSE-Cog**	**Pre-treatment**		**22.2**	0.5		16.8		2.9	27.0	30					
		**Post-treatment**		**23.9**	0.5		18.0		24.5	28.0	30					
		**Difference**		**1.7**	0.3		--		--	--	30	<0.001				
**Zhao et al. (2017)** [34]	**Neuropsychological assessments**	**Group (N)**			**Mean scores (SD)**					***p*-value**						
					**Baseline [B]**	**Immediate after treatment [6]**			**6 weeks after treatment [12]**	**[B] vs. [6]**		**[B] vs. [12]**	Time * group			
	**ADAS-cog**	**Treatment (17)**			22.6 (5.9)	18.5 (5.4)			16.8 (6.9)	0.042 *		0.013 *	0.332			
		**Sham (13)**			24.2 (6.1)	22.9 (8.9)			21.2 (8.6)	0.668		0.315				
		**Mild treat. (12)**			20.6 (5.2)	16.4 (4.4)			14.2 (6.8)	0.044 *		0.017 *	0.263			
		**Mild sham (8)**			21.7 (4.6)	20.3 (5.6)			19.4 (6.8)	0.593		0.441				
		**Moderate treat. (5)**			23.8 (6.6)	20.3 (6.5)			18.9 (6.7)	0.423		0.278	0.878			
		**Moder sham (5)**			27.5 (5.9)	24.2 (8.6)			23.5 (8.5)	0.499		0.413				
	**MMSE**	**Treatment (17)**			22.2 (2.8)	23.9 (2.5)			25.5 (4.6)	0.071		0.017 *	0.557			
		**Sham (13)**			22.8 (2.3)	23.1 (3.3)			24.2 (4.1)	0.790		0.294				
		**Mild treat. (12)**			25.6 (2.1)	27.1 (4.1)			29.7 (4.5)	0.147		0.042	0.639			
		**Mild sham (8)**			25.8 (2.3)	26.2 (3.5)			28.1 (3.5)	0.791		0.395				
		**Moderate treat. (5)**			19.2 (2.5)	20.4 (3.3)			21.7 (4.3)	0.535		0.294	0.812			
		**Moderate sham.(5)**			19.5 (1.9)	20.4 (2.5)			21.5 (2.1)	0.539		0.153				
	**MoCA**	**Treatment (17)**			17.5 (6.2)	19.8 (6.5)			21.5 (5.9)	0.299		0.063	0.552			
		**Sham (13)**			18.1 (7.3)	19.3 (6.7)			20.1 (6.6)	0.666		0.471				
		**Mild treat. (12)**			18.6 (5.1)	21.1 (4.3)			23.1 (5.3)	0.208		0.046 *	0.799			
		**Mild sham (8)**			19.7 (7.5)	20.9 (7.1)			21.4 (7.8)	0.747		0.664				
		**Moderate treat. (5)**			16.6 (6.2)	17.9 (5.8)			19.2 (5.5)	0.741		0.503	0.517			
		**Moder sham (5)**			17.5 (6.8)	18.4 (5.9)			19.5 (6.6)	0.829		0.650				
	**WHO-UCLA AVLT**	**Treatment (17)**			32.5 (7.9)	35.8 (7.8)			38.7 (8.9)	0.229		0.039 *	0.667			
		**Sham (13)**			34.1 (8.1)	35.8 (7.4)			37.8 (8.7)	0.582		0.273				
		**Mild treat.(12)**			35.6 (5.6)	37.9 (6.5)			41.8 (6.6)	0.363		0.021 *	0.524			
		**Mild sham (8)**			35.8 (6.7)	36.6 (6.7)			38.7 (4.5)	0.815		0.327				
		**Moderate treat.(5)**			30.5 (7.6)	33.5 (2.3)			35.6 (6.3)	0.423		0.281	0.550			
		**Moder sham (5)**			30.6 (6.7)	33.9 (5.4))			36.5 (4.9)	0.416		0.151				
	**Group**	**Cognitive domains**			**Changes in score, mean (SD)**					***p*-value**						
					**Δ immediate after treatment**				**Δ 6 weeks after treatment**	**[B] vs. [6]**		**[B] vs. [12]**	Time * group			
	**Treatment**	**Memory**			2.04 (2.25)				2.62 (3.66)	0.002 *		0.009 *	0.575			
		**Language**			1.17 (1.36)				1.39 (1.67)	0.003 *		0.003 *	0.676			
		**Executive function**			0.86 (1.89)				0.49 (1.67)	0.079		0.244	0.549			
	**Mild treatment**	**Memory**			2.56 (2.33)				2.88 (2.87)	0.003 *		0.005 *	0.767			
		**Language**			1.36 (1.56)				1.54 (1.67)	0.012 *		0.009 *	0.788			
		**Executive function**			0.92 (1.12)				0.92 (1.50)	0.016 *		0.058	1.000			
	**Moderate treatment**	**Memory**			1.56 (2.32)				2.41 (3.12)	0.207		0.159	0.638			
		**Language**			0.98 (0.89)				1.15 (1.56)	0.070		0.175	0.838			
		**Executive function**			0.74 (0.99)				0.00 (1.11)	0.170		1.000	0.298			
**Nguyen et al. (2017)** [35]					**Baseline**				**Post**	**Follow-up 6 months**			***p***			
	**ADAS-Cog**				20.1 (3.1)				17.2 (2.5)	20.1 (3.4)			0.0165			
	**ADAS-Cog word recognition**				17.8 (1.4)				18.5 (1.7)	19.0 (1.3)			0.1738			
	**ADAS-Cog word recall**				2.7 (0.5)				2.8 (0.5)	2.7 (0.5)			0.9592			
	**MMSE**				18.8 (1.9)				19.7 (1.4)	17.8 (1.5)			0.1168			
	**MMSE language**				6.4 (0.4)				6.6 (0.3)	6.6 (0.4)			0.5945			
	**Dubois**				5.0 (0.8)				4.3 (0.85)	4.5 (1.1)			0.2466			
	**FAB**				11.5 (1.3)				11.7 (1.4)	11.4 (1.5)			0.9155			
	**Stroop color test**				39.3 (7.3)				36.3 (6.1)	40.6 (8.2)			0.8357			
	**Locomotor**				26.2 (0.6)				27.0 (0.4)	27.0 (0.5)			0.0478			
	**Apathy**				17.4 (2.7)				10.8 (2)	9.4 (1.8)			0.0125			
	**Caregiver (Zarit)**				4.1 (0.4)				3.6 (0.5)	3.7 (0.4)			0.2385			
	**Dependence**				48.4 (5.5)				36.8 (5)	34.7 (4.4)			0.0085			
**Sabbagh et al. (2019)** [30]		**Baseline**							**Post (week 7)**				**Post (week 12)**			
		**AG**			**SG**				**AG**	**SG**			**AG**	**SG**		
	**ADAS**	23.6			24.4				−0.7	-0.7			−2.11	24.4		
	**MMSE**	21.7			21.3				N/A	N/A			N/A	N/A		
**Zhang et al.** **(2019)** [33]	**Neuropsychological assessment**		**Sham rTMS-CT** **(Mean ± SE)**					**Real rTMS-CT** **(Mean ± SE)**		**F value**			***p-*** **value**			
	**MMSE**															
	[4]-[B]		0.75 ± 0.49					3.27 ± 0.56		17.411			<0.01 *			
	[8]-[B]		0.80 ± 0.81					2.571 ± 0.45		5.894			0.024 *			
	**ACE-III**															
	[4]-[B]		2.18 ± 1.43					11.77 ± 1.32		25.817			<0.01 *			
	[8]-[B]		0.69 ± 1.63					7.70 ± 1.51		10.692			<0.01 *			
	**Attention**															
	[4]-[B]		0.07 ± 0.49					2.19 ± 0.44		19.308			<0.01 *			
	[8]-[B]		−0.14 ± 0.53					1.88 ± 0.40		22.590			<0.01 *			
	**Memory**															
	[4]-[B]		0.29 ± 1.07					3.87 ± 0.82		7.798			0.009 *			
	[8]-[B]		−0.36 ± 1.22					1.87 ± 0.63		4.021			0.055			
	**Verbal fluency**															
	[4]-[B]		0.21 ± 0.42					0.94 ± 0.42		2.951			0.097			
	[8]-[B]		0.14 ± 0.43					0.69 ± 0.36		3.208			0.084			
	**Language**															
	[4]-[B]		1.00 ± 0.92					2.31 ± 0.89		3.310			0.080			
	[8]-[B]		1.71 ± 0.79					2.25 ± 0.44		2.697			0.113			
	**Visuospatial function**															
	[4]-[B]		0.79 ± 0.65					1.56 ± 0.45		5.472			0.027 *			
	[8]-[B]		0.57 ± 0.61					1.56 ± 0.43		7.416			0.011 *			
			**PRE**					23.00 (2.48)		25.10 (3.06)			23.61 (3.99)			
			**POST**					20.79 (2.81)		24.7 (3.23)			24.11 (3.31)			

AG: active group; SG: sham group; Sd: standard deviation; N/A: not available; N: number of participants; MMSE: Mini Mental Stage Test; ADAS-cog: Alzheimer Disease Assessment Scale—Cognitive; CGIC: Clinical Global Impression of Change scale; ADAS-ADL: Alzheimer Disease Assessment Scale—Activities of Daily Living; Hamilton Rating Scale for Depression; NPI: Neuropsychiatric Inventory test; ICF, intracortical facilitation; FAB: Frontal Assessment Battery; Dubois score; WHO-UCLAAVLT: World Health Organization University of California—Los Angeles, Auditory Verbal Learning Test; ACE-III: Addenbrooke’s Cognitive Examination III.

## Data Availability

No new data were created.

## Appendix A

**Section and Topic**	**Item #**	**Checklist Item**	**Location Where Item Is Reported**
TITLE			
Title	1	Identify the report as a systematic review.	1
ABSTRACT			
Abstract	2	See the PRISMA 2020 for Abstracts checklist.	1
INTRODUCTION			
Rationale	3	Describe the rationale for the review in the context of existing knowledge.	1, 2
Objectives	4	Provide an explicit statement of the objective(s) or question(s) the review addresses.	1, 2
METHODS			
Eligibility criteria	5	Specify the inclusion and exclusion criteria for the review and how studies were grouped for the syntheses.	2
Information sources	6	Specify all databases, registers, websites, organizations, reference lists, and other sources searched or consulted to identify studies. Specify the date when each source was last searched or consulted.	3
Search strategy	7	Present the full search strategies for all databases, registers, and websites, including any filters and limits used.	3
Selection process	8	Specify the methods used to decide whether a study met the inclusion criteria of the review, including how many reviewers screened each record and each report retrieved, whether they worked independently, and, if applicable, details of automation tools used in the process.	4
Data collection process	9	Specify the methods used to collect data from reports, including how many reviewers collected data from each report, whether they worked independently, any processes for obtaining or confirming data from study investigators, and, if applicable, details of automation tools used in the process.	4
Data items	10a	List and define all outcomes for which data were sought. Specify whether all results that were compatible with each outcome domain in each study were sought (e.g., for all measures, time points, analyses), and if not, the methods used to decide which results to collect.	NA
	10b	List and define all other variables for which data were sought (e.g., participant and intervention characteristics, funding sources). Describe any assumptions made about any missing or unclear information.	NA
Study risk of bias assessment	11	Specify the methods used to assess the risk of bias in the included studies, including details of the tool(s) used, how many reviewers assessed each study and whether they worked independently, and if applicable, details of automation tools used in the process.	4, 5
Effect measures	12	Specify for each outcome the effect measure(s) (e.g., risk ratio, mean difference) used in the synthesis or presentation of results.	NA
Synthesis methods	13a	Describe the processes used to decide which studies were eligible for each synthesis (e.g., tabulating the study intervention characteristics and comparing against the planned groups for each synthesis (item #5)).	2
	13b	Describe any methods required to prepare the data for presentation or synthesis, such as handling of missing summary statistics or data conversions.	NA
	13c	Describe any methods used to tabulate or visually display the results of individual studies and syntheses.	
	13d	Describe any methods used to synthesize results and provide a rationale for the choice(s). If meta-analysis was performed, describe the model(s), method(s) to identify the presence and extent of statistical heterogeneity, and software package(s) used.	NA
	13e	Describe any methods used to explore possible causes of heterogeneity among study results (e.g., subgroup analysis, meta-regression).	NA
	13f	Describe any sensitivity analyses conducted to assess the robustness of the synthesized results.	NA
Reporting bias assessment	14	Describe any methods used to assess the risk of bias due to missing results in a synthesis (arising from reporting biases).	NA
Certainty assessment	15	Describe any methods used to assess certainty (or confidence) in the body of evidence for an outcome.	NA
RESULTS			
Study selection	16a	Describe the results of the search and selection process, from the number of records identified in the search to the number of studies included in the review, ideally using a flow diagram.	NA
	16b	Cite studies that might appear to meet the inclusion criteria but which were excluded, and explain why they were excluded.	NA
Study characteristics	17	Cite each included study and present its characteristics.	9
Risk of bias in studies	18	Present assessments of risk of bias for each included study.	NA
Results of individual studies	19	For all outcomes, present, for each study, (a) summary statistics for each group (where appropriate) and (b) an effect estimate and its precision (e.g., confidence/credible interval), ideally using structured tables or plots.	17–25
Results of syntheses	20a	For each synthesis, briefly summarise the characteristics and risk of bias among contributing studies.	NA
	20b	Present results of all statistical syntheses conducted. If meta-analysis was performed, present for each the summary estimate and its precision (e.g., confidence/credible interval) and measures of statistical heterogeneity. If comparing groups, describe the direction of the effect.	NA
	20c	Present results of all investigations of possible causes of heterogeneity among study results.	NA
	20d	Present results of all sensitivity analyses conducted to assess the robustness of the synthesized results.	NA
Reporting biases	21	Present assessments of risk of bias due to missing results (arising from reporting biases) for each synthesis assessed.	NA
Certainty of evidence	22	Present assessments of certainty (or confidence) in the body of evidence for each outcome assessed.	NA
DISCUSSION			
Discussion	23a	Provide a general interpretation of the results in the context of other evidence.	26, 27
	23b	Discuss any limitations of the evidence included in the review.	26, 27
	23c	Discuss any limitations of the review processes used.	27
	23d	Discuss the implications of the results for practice, policy, and future research.	27
OTHER INFORMATION			
Registration and protocol	24a	Provide registration information for the review, including the register name and registration number, or state that the review was not registered.	NA
	24b	Indicate where the review protocol can be accessed or state that a protocol was not prepared.	NA
	24c	Describe and explain any amendments to information provided at registration or in the protocol.	NA
Support	25	Describe sources of financial or non-financial support for the review and the role of the funders or sponsors in the review.	27
Competing interests	26	Declare any competing interests of review authors.	27
Availability of data, code, and other materials	27	Report which of the following are publicly available and where they can be found: template data collection forms; data extracted from included studies; data used for all analyses; analytic code; any other materials used in the review.	27
